# Emergence of Bictegravir Resistance in a Treatment-Experienced PWH on Functional Monotherapy and Rapid Replacement by an Ancient Wild-Type Strain Following Transient Treatment Interruption

**DOI:** 10.3390/v17050699

**Published:** 2025-05-13

**Authors:** Pietro B. Faré, Gabriela Ziltener, Judith Bergadà Pijuan, Irene A. Abela, Britta L. Hirsch, Michael Huber, Johannes Nemeth, Huldrych F. Günthard

**Affiliations:** 1 Department of Infectious Diseases and Hospital Epidemiology, University Hospital Zurich, 8091 Zurich, Switzerland; 2 Institute of Medical Virology, University of Zurich, 8057 Zurich, Switzerland

**Keywords:** HIV-1, antiretroviral therapy, bictegravir, virological failure, drug resistance, M184V/I mutation, integrase strand transfer inhibitor, ART interruption, wild-type re-emergence

## Abstract

A treatment-experienced, highly adherent person living with HIV for over 25 years developed resistance mutations against all four major ART classes, including bictegravir (BIC). This led to viral failure on a quadruple regimen including BIC and doravirine (DOR). Resistance emergence was associated with M184V, thymidine analog mutations (TAMs), NNRTI mutations (108I, 234I, 318F), and INSTI mutations (T97A, G140S, Q148H, G149A), likely driven by suboptimal BIC levels due to divalent cation interactions. During a two-month ART interruption, the resistant virus was rapidly replaced by an ancient wild-type strain. Despite resistance to all four ART classes, a genotype-adapted salvage regimen, including fostemsavir, achieved viral suppression within seven months.

## 1. Case Report

A 27-year-old male was diagnosed with HIV-1 infection Subtype B in 1994. At that time, he was enrolled in the Swiss HIV Cohort Study [1]. At diagnosis, HIV-1 viral load (VL) was 115,000 copies/mL, with a nadir CD4+ cell count of 200 cells/µL (9%). Zidovudine (AZT) and ritonavir (RTV) were initiated about a year later. At that time, the only HIV-related complication was oral leukoplakia.

In 1996, due to never having reached a suppressed plasma viral load, a first genotypic resistance test on HIV-1 plasma RNA was performed and showed the mutation K70R associated with thymidine analog nucleoside (TAM) reverse transcriptase inhibitor (NRTIs) resistance and a pattern of mutations (I54V, V82AT) associated with resistance to all protease inhibitors (PIs) and increased susceptibility to darunavir (DRV).

**Figure 1:**
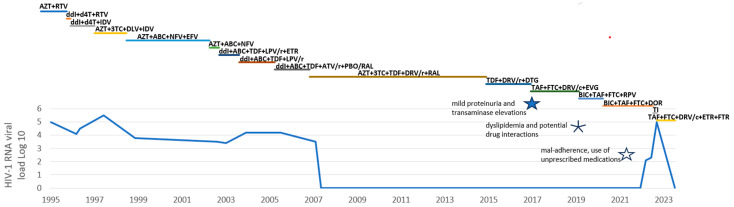
HIV-1 VL (logarithmic scale) and ART combinations over time. AZT = Zidovudine, ddI = Didanosine, d4T = Stavudine, RTV = Ritonavir, IDV = Indinavir, 3TC = Lamivudine, DLV = Delavirdine, ABC = Abacavir, NFV = Nelfinavir, EFV = Efavirenz, TDF = Tenofovir disoproxil fumarate, LPV/r = Lopinavir boosted with Ritonavir, ETR = Etravirine, ATV/r = Atazanavir boosted with Ritonavir, PBO/RAL = Placebo or Raltegravir, DRV/r = Darunavir boosted with Ritonavir, RAL = Raltegravir, DRV/c = Darunavir boosted with Cobicistat, DTG = Dolutegravir, FTC = Emtricitabine, EVG = Eviltegravir, BIC = Bictegravir, RPV = Rilpivirine, DOR = Doravirine, TI = Treatment Interruption, FTR = Fostemsavir.

During the first 8 years of treatment, antiretroviral therapy (ART) could only partially control HIV-1, with persistent viremia despite multiple different combinations of ART (Figure 1). Thus, in 2002 another genotypic resistance test was performed and revealed the additional TAM D67N, in addition to the K70R already described. PI mutations were unchanged.

In 2003, in the context of the TMC-125 trial, ART was switched to didanosine (ddI), abacavir (ABC), tenofovir disoproxil (TDF), lopinavir/ritonavir (LPV/r), and etravirine (ETR). This combination of drugs again failed to achieve viral suppression, as did the combination ddI/ABC/TDF and atazanavir/ritonavir (ATV/r) in 2005. Viral suppression first occurred in 2007 with the introduction of the integrase strand transfer inhibitor (INSTI) raltegravir (RAL) and the switch from ATV/r to DRV/r in combination with AZT/3TC and TDF. From then on, HIV-1 VL was fully suppressed below the limit of quantification of 50 copies/mL plasma, with CD4+ cell counts ranging from 690 cells/µL to 720 cells/µL. Treatment adherence was consistently reported to be optimal [2]. A DNA-based genotypic resistance assay on proviral DNA in 2015, within the framework of the Clinical Trial CN518, revealed the additional emergence of the NRTI resistance mutation M184V. This led the treating team to discontinue AZT/3TC, while continuing TDF. At the same time, RAL was replaced by the meanwhile available dolutegravir (DTG) to strengthen the genetic resistance barrier. Two years later, in 2017, the therapy was switched to tenofovir alafenamide, emtricitabine, elvitegravir, in a single tablet regimen with the booster cobicistat (TAF/FTC/ELV/c) and DRV. Over the last ten years, the patient has manifested chronic primary headache and a depressive syndrome with insomnia, which eventually required a therapy with trazodone and zolpidem, a metabolic syndrome with grade 1 obesity, type 2 diabetes, and dyslipidemia. In summer 2019, due to concerns about dyslipidemia and potential interactions with cobicistat, trazodone, and zolpidem, DRV was stopped, and the ART regimen was further modified to BIC/TAF/FTC and the NNRTI rilpivirine (RPV). During this period, therapeutic adherence was assessed every 3 months and always reported to be excellent: the patient regularly took prescribed medication and attended all medical appointments. Nevertheless, he began to self-medicate by taking medicines and supplements without a doctor’s prescription, often buying them over the counter or online rather than from a pharmacy. Moreover, the communication with caregivers about the use of these drugs and supplements was not always straightforward. As part of his bodybuilder nutritional regime, he began taking a phytotherapeutic preparation (Tribulus terrestris, undefined dosage, at body-builder training sessions, about four times a week) known for its effects on the sex hormone axis. In the same period, without informing the healthcare team, he started taking zinc (zinc picolinate 30 mg once daily), iron (preparation, dose, and frequency not further specified), and magnesium supplements (magnesium aspartate 10 mmol once or twice daily), poly-vitamins containing the same divalent cations and an over-the-counter antacid containing calcium (calcium carbonate, and natrium alginate suspension, on demand) magnesium and aluminum (aluminum hydroxide and magnesium hydroxide suspension, on demand).

Considering the patient’s concerns about medical therapy for gastric acid reduction, ART was modified in August 2020 by introducing doravirine (DOR) instead of RPV due to the latter’s potential interaction with proton pump inhibitors. Regrettably, due to digitalization of the original paper charts, resulting in more difficult access of old chart data, including resistance tests, it was overseen that between 1999 and 2006 the selection of additional NNRTI resistance-associated mutations (RAMs) had occurred: K108I, L234I, and Y318F, the latter two associated with resistance to DOR. These three mutations were probably acquired due to previous viral failures with NNRTIs: in 1998, during a clinical trial with delavirdine and in 2002, with a combination therapy with efavirenz (Figure 1).

In July 2022, for the first time since 2007, we noted a detectable HIV-1 VL of 134 copies/mL. After additional three months of DOR plus BIC/TAF/FTC, the HIV-1 VL rose to 187 copies/mL. Therapeutic adherence was strictly monitored, and it was found that the patient was not following the correct interval between divalent cation containing drugs and BIC. The therapeutic drug monitoring trough level of DOR at this time point was within the normal range (45th percentile), whereas the trough level for BIC was reduced to below the 5th percentile. This pattern showed that the patient regularly took the drugs but due to pharmacological interactions with the divalent cations the BIC level was too low.

Close monitoring of the HIV-1 VL after three weeks showed a still elevated viral load of 217 copies/mL. A genotypic resistance test was carried out, which showed emergence of resistance to all the INSTIs, including BIC (Table 1).

Retrospective genotypic resistance tests were performed on proviral DNA obtained from peripheral blood mononuclear cells (PBMCs) of the Swiss HIV Cohort Study biobank [1] taken seven months earlier (with HIV-1 VL < 20 copies/mL) and on biobanked plasma (with HIV-1 VL 134 copies/mL) taken four months earlier. These tests showed that the newly acquired resistance pattern has been acquired between April 2022 and July 2022 and was already present at the first detectable viral load (VL 134 copies/mL) after being suppressed for >15 years.

Based on the patient’s history and current resistance pattern, we designed a new drug regimen: replacing BIC/FTC/TAF and DOR with FTC/TAF/DRV/c plus ETR (the only NNRTI still active) and fostemsavir (FTR), a new attachment inhibitor specifically approved for multi-drug-resistant HIV-1 infection in combination with an optimized backbone therapy. Pending the health insurance’s response for FTR reimbursement, the patient, psychologically destabilized by the situation, informed us that he was going to have a trip abroad to recover. In this context, the ongoing therapy was completely suspended.

At the next follow-up two months later, the HIV-1 VL had increased to 106,000 copies/mL, CD4+ cells were 566 cells/µL. A genotypic resistance test [3] using next generation sequencing showed a complete “wild type” for all RAMs with a sensitivity cut-off of 1.5% at a minimum depth of 1750 reads.

**Figure 2:**
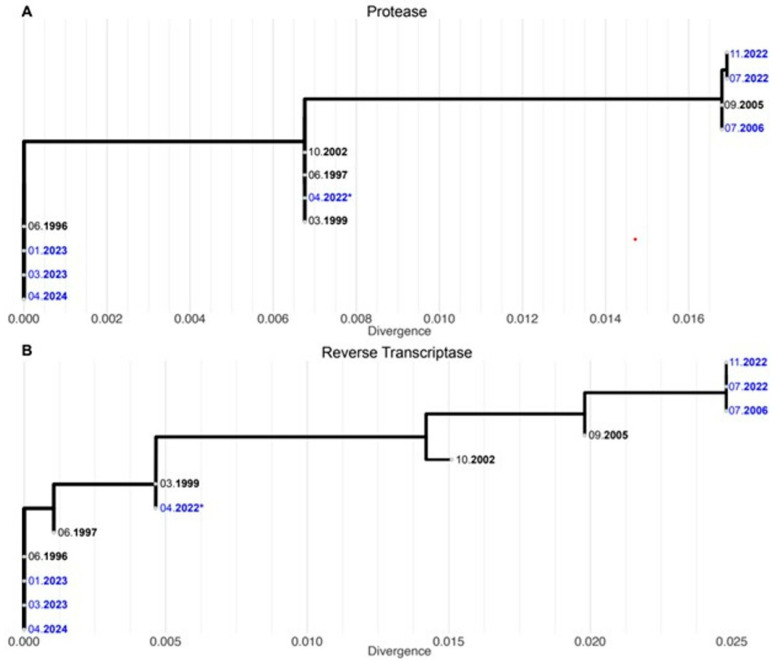
Maximum likelihood (ML) phylogenetic tree showing evolution in the HIV-1 (**A**) protease and (**B**) reverse transcriptase, excluding drug resistance mutations. The X-axis shows the divergence of the strains (i.e., nucleotide changes per site). Samples reported in Table 1 are marked in blue. Asterisk indicates proviral DNA sequence. We accounted only for changes in the viral background, omitting drug resistance mutations. Sequences were aligned using MAFFT v7.526 (with—auto option) [4]. The ML phylogenetic tree was created using Nextstrain augur version 8.5.484 [5]. The tree was refined providing information on the dates of the different strains with the option—metadata, and the ancestral sequences were inferred keeping the ambiguous nucleotides (—keep-ambiguous). The resulting trees were exported for visualization with Nextstrain auspice (with the option—include-root-sequence).

To determine whether this observation was rather a replacement of the resistant strain by an ancient archived wild-type or reversions at the specific resistance associated nucleotide positions, all available sequences of the HIV-1 protease and reverse transcriptase were compared. This phylogenetic analysis revealed that the wild-type sequence was an ancient archived virus strain, which most likely reemerged from the latent reservoir in the absence of any selective drug pressure after the treatment stopped, fully replacing the resistant strain (Figure 2).

The new ART regimen was started as planned, when the patient came back from his vacation. HIV-1 VL decreased steadily but relatively slowly, with achieving non-detectability only after 7 months of treatment. To identify potentially selected RAMs after initiation of the new drug regimen, a further genotypic resistance test was conducted two months after the start of the new antiretroviral therapy at HIV-1 RNA VL of 1590 copies/mL of plasma. The result again showed only the wild-type virus. During the subsequent period, except for a brief episode of nonadherence, the patient fully adhered to the new ART regimen, which was generally well tolerated and led to a sustained suppression of the HIV-1 viral load. The residual drug levels of DRV/r, TAF, and FTC remained within the normal range.

## 2. Discussion

We describe a rare case of virological failure on a quadruple therapy with BIC/3TC/TAF and DOR in a treatment-experienced patient. INSTI resistance-associated mutations emerged, conferring resistance also to bictegravir. Interestingly, after a transient interruption of antiretroviral therapy, we were able to document the reappearance of an ancient archived wild-type virus rapidly replacing the resistant virus, as determined by sequence analysis, probably due to the high fitness cost of the accumulated drug resistance mutations.

Integrase strand transfer inhibitors are recommended in initial antiretroviral therapy for HIV-1 infection [6,7]. Second-generation INSTIs, bictegravir, and dolutegravir, are preferred for their efficacy, tolerability, and high resistance barriers. Due to these characteristics, bictegravir, in the single tablet regimen BIC/3TC/TAF, is considered also as one of the best choices in treatment-experienced patients. The BRAAVE study, a recent phase 3b, multicenter, open-label study, demonstrated the efficacy of switching to BIC/3TC/TAF in suppressed individuals with maintenance of virologic suppression for 72 weeks in 99% of eligible individuals, regardless of pre-existing resistance, viral blips, and suboptimal adherence [8].

The emergence of bictegravir RAMS is rare in ART-naïve PWH (<1%) and in PWH previously treated with ART [9,10,11]. A recent French retrospective observational study reported a 6.8% prevalence of virological failure among individuals receiving BIC/FTC/TAF as first- or second-line therapy; among these cases, emergent INSTI resistance-associated mutations (RAMs) were detected in 4.2% of patients [12].

In our patient, virological failure was probably triggered by several factors, culminating in bictegravir resistance.

(i) The patient harbored the M184V mutation, which confers a high level of resistance to FTC. Notwithstanding, M184V mutation in itself is not considered a contraindication to continue treatment with FTC and TAF or TDF, because it increases susceptibility to tenofovir. However, the co-expression of TAMs (thymidine analog-associated mutations) D67N and K70R may have led to a potential low-level resistance to tenofovir, limiting the efficacy of this NRTIs combination. Indeed, it has been shown that the accumulation of at least two TAMs results in reduced susceptibility to tenofovir, thereby reducing the resensitizing effect of M184V against tenofovir [13].

(ii) About two years before virologic failure, rilpivirine had been replaced by doravirine because of the former’s interaction with proton pump inhibitors. Co-prescription of BIC/3TC/TAF and DOR has been shown to be highly effective and safe even in heavily treatment-experienced individuals, as shown in a recent open-label switch trial [14]. Cross-resistance between doravirine and other NNRTIs is nonetheless common, particularly in individuals who fail on NNRTI regimens [15,16]. Previous failures with other NNRTIs or known NNRTI mutations should be critically considered before switching to doravirine. Our patient failed delavirdine and efavirenz and developed NNRTI RAMs K108I, L234I, and Y318F, the latter two associated with resistance to doravirine. He was therefore unsuitable for switching to doravirine.

(iii) The undeclared use of magnesium, iron, zinc, multivitamins, and over-the-counter antacids containing aluminum, calcium, and magnesium taken concomitantly with bictegravir resulted in a predictable reduction in the peak-and-trough plasma concentrations of the drug (measured below the 5th percentile at the time of treatment failure). From a pharmacokinetic point of view, it is interesting to note that the patient was fully adherent with regard to tablet-taking (as evidenced by the concomitant 35% percentile trough plasma level of doravirine). We believe that these circumstances (functional INSTI monotherapy and reduced BIC plasma concentrations) created the ideal conditions for the emergence of new INSTI RAMs, somehow confirming the observation that second-generation INSTIs (DTG and BIC) may have a lower resistance barrier than postulated when administered with a less than fully active backbone comedication [17,18].

After just two months of the unplanned ART pause, HIV-1 viral load surged, and an ancient wild-type virus quickly reappeared, as shown by sequence analysis. All drug resistances so far documented by RNA and proviral DNA sequencing in all four main ART classes (D67N, K70R, M184V for NRTIs, 108I, 234I, 318F for NNRTIs, L10V, K20R, T74S for PIs and T97A, G140S, Q148H, G149A for INSTIs) were completely undetectable at a very low frequency threshold. A phylogenetic analysis of the HIV-1 resistance-associated regions PR (protease) and RT (reverse transcriptase) confirmed that the re-emerged virus strain was an ancient archived wild type.

To the best of our knowledge, this is the first report in the literature to clearly document the rapid replacement of a resistant strain harboring resistance-associated mutations in all four major classes of ART drugs (NRTIs, NNRTIs, PIs, and INSTIs). This finding underscores the severe fitness cost associated with these mutations, most likely also caused by the INSTI-associated resistance mutations [19].

The direct impact INSTI RAMs on viral replication is a matter of study: the Q148H mutation alone significantly impairs viral replication by disrupting the integrase enzyme’s catalytic activity, leading to reduced efficiency in the integration of viral DNA into the host genome. However, when G140S co-occurs with Q148H, it can partially compensate for this replication defect, restoring viral replication capacity closer to wild-type levels. The T97A mutation has minimal impact on replication capacity, while the specific impact of G149A on viral replication is less well characterized and warrants further investigation.

The suggested strong in vivo fitness cost of the INSTI RAMs as observed in our case may be an explanation that so far globally hardly any transmitted INSTI RAMs have been detected. However, systematic additional population-based drug resistance screenings will be needed to determine a potential contribution of transmitted INSTI drug resistance to treatment response in the future [20].

This case report provides rare, well-documented insights into the development of bictegravir resistance and the rapid re-emergence of archived wild-type virus following interruption of antiretroviral therapy. Its strengths include pharmacokinetic analysis, comprehensive resistance testing, exact drug and adherence history, next-generation sequencing from HIV-1 RNA and DNA and phylogenetic analysis. A potential limitation of this study is the absence of data on viral populations in anatomical compartments beyond the bloodstream, as resistant HIV variants may persist in latent reservoirs and re-emerge under selective drug pressure, thereby complicating long-term therapeutic outcomes.

The emergence of resistance due to subtherapeutic drug levels—resulting from drug–drug or drug–nutrient interactions—or the use of potentially ineffective regimens raises ethical concerns regarding informed patient care and access to relevant medical data. Clinicians are ethically obligated to ensure optimal drug absorption, monitor for resistance, minimize harmful interactions, and select therapies tailored to the patient’s history and prior treatments. Failure to address these factors can undermine treatment efficacy, accelerating resistance, which is especially critical in chronic conditions like HIV.

## Figures and Tables

**Table 1:** Summary of genotypic resistance tests (on RNA or proviral DNA) and relevant mutations and selected additional plasma RNA measurements.

Date	VL Copies/mL	Ongoing ART for Each Time Interval by Class					Resistance Test	Relevant Resistance Associated Mutations by ART Class			
		NRTI	PI	NNRTI	INSTI	Other		NRTI	NNRTI	PI	INSTI
07.2006	14,500	ddI + ABC + TDF	ATV/r		RAL		RNA	D67N, K70R	V108I, L234I, Y318 F	L10I, K20R, K43T, M46L, I54V, V82M, L90M	not done
04.2022	<20	TAF + FTC		DOR	BIC		proviral DNA (PBMC)	D67N, K70R	V108I, L234I, Y318 F	L10I, K20R, K43T, M46L, I54V, V82M, L90M	none
07.2022	134	TAF + FTC		DOR	BIC		RNA	D67N, K70R	V108I, L234I, Y318 F	L10I, K20R, K43T, M46L, I54V, V82M, L90M	T97A, G140S, Q148H
11.2022	217	TAF + FTC		DOR	BIC		RNA	D67N, K70R, M184V	V108I, L234I, Y318 F	L10I, K20R, K43T, M46L, I54V, V82M, L90M	T97A, G140S, Q148H, G149A
01.2023	106,000	TAF + FTC	DRV/c	ETR		FTR	RNA	none	none	none	none
03.2023	1590	TAF + FTC	DRV/c	ETR		FTR	RNA	none	none	none	none
07.2023	<20	TAF + FTC	DRV/c	ETR		FTR	not done	not done	not done	not done	not done
04.2024	3900	TAF + FTC	DRV/c	ETR		FTR	RNA	none	none	none	none
07.2024	<20	TAF + FTC	DRV/c	ETR		FTR	not done	not done	not done	not done	not done

## Data Availability

The data that support the findings of this case report are not publicly available due to concerns regarding patient privacy and confidentiality. Data are available from the corresponding author upon reasonable request and with appropriate institutional approvals.
